# Dental Education

**Published:** 1854-07

**Authors:** J. S. Rock

**Affiliations:** Dentist.


### ARTICLE XVI.

*Dental Education.* By J. S. Rock, M. D., Dentist.

The importance of a liberal education to the practicing dentist, is a subject which we think has been too much overlooked by the profession. Every intelligent person is ready to admit that all professional men should be liberally educated—while the dentist, an exception to the rule, if he has learned a few technical terms from some anatomical work, possesses the strength of a blacksmith, the mechanical ingenuity of a tinker, and a flippant tongue, goes forth with a few "specimens" (made by some journeyman,) as a scientific dentist, when, in fact, he cannot distinguish between the normal and abnormal condition of the tissues, and in the majority of cases, if called upon, could not discriminate between neuralgia and odontalgia.

Dentistry has always been too much looked upon as a trade, in which mechanical talent is alone called into action, and only a moderate share of that; the result is, too many have been induced to enter into it with the (vain) hope of learning all there is to be learned in a few months, at a trifling expense, with the expectation of a fortune in a short time. This ambitious desire to make money, at the expense of life, or limb, or both, is much to be deprecated. A man who has no higher motive than money-making, is unfit to be placed in so responsible a position as that of a dentist or a physician.

We have dental societies and colleges now, and we expect to see, ere long, a distinction between the scientific dentist, and the semi-ignorant practitioner. It will take time, patience and perseverance, on the part of the profession to accomplish this end, and these institutions will do much to forward it; able men are in the work, and it must succeed. Then, and not till then, will dentistry be exalted to that position which the scientific portion of the profession are aiming for, and which it is destined sooner or later to attain.

Our plan is, that young men, before entering the profession, shall finish their English and classical education; for we assure them that a sound classical education will open stores of learning to them, which are sealed books to others. Technical terms abound very luxuriantly in the different branches of our science, and these have nearly all been derived from the classics. Their acquisition is always a matter of difficulty to the English scholar, but that difficulty is much lessened if the student is familar with the Latin and Greek. They will find that there is a great deal to be learned from the ancient authors, far more than enough to encourage them to read them in their original tongues.

There are many valuable works written on surgery, chemistry and dentistry, by the French and Germans, and we lose much valuable information if we are unable to read these languages. A general knowledge of the sciences is of great importance to the dental surgeon, and will furnish him with invaluable assistance in diagnosing. The object of all science is to discover facts and trace their relations; and unless we are acquainted

53*

with them, our judgment must be limited. They strengthen our minds and enable us to determine what any series of effects and causes will have upon each other, which is our great duty in investigating the cause of disease, and the application of the remedy.

We should also gain by every means an accurate knowledge of facts, and enlarge their number to the utmost possible extent; and beyond this, we should strive to obtain a habit of discerning the dependence of facts upon each other, and the whole upon general principles.

The mechanics of the human body is a subject of great interest, and will furnish the most perfect models for imitation; the varied action ef the muscles are in harmony with the action of levers universally, for these laws have been deduced from the operations of nature, of which we have the most perfect illustration.

The science of acoustics is an invaluable aid to us in the physical examination of the lungs, heart, &c., and we now look to that science to resolve certain doubts which still envelop the subject.

Optics is another important aid. The benefits resulting from the use of the microscope in the study of anatomy and physiology, and the practice of medicine, is truly astonishing. A distinguished London surgeon says, "the smallest portion of a diseased structure, placed on the field of the microscope, will tell more to the experienced eye in one minute, than could be acquired from a week's examination of the crude mass of disease, as preserved in any museum." The same may be said of other branches of science.

The different temperaments, habits and modes of living are objects of vast importance, and should be well understood. The same medicine will often produce different effects upon different constitutions. *Exegesis*, the administration of an emetic in one constitution will produce free emesis, while in another it may produce violent cramp in the stomach. Again, a cathartic given on an empty stomach will generally purge freely, but if given after a full meal will arrest digestion, and often produce nausea and vomiting. A slight wound in one person may heal by the first intention, while in another it may result in traumaic tetanus and death!

A general knowledge of the different branches of medical science is of infinite importance to the dentist, and without it he may not expect to excel. The sciences of medicine and dentistry are so intimately connected with each other, that to separate the one from the other is to clip the branches from the trunk. The treatment of all diseases of the mouth, and the performance of all operations in that cavity, belong peculiarly to the dental surgeon; but if he is ignorant of said branches, it would be extremely dangerous for him to operate for a cleft palate, to extract a tumor, or to excise a superior or inferior maxilla, especially when we consider the difficulties which attend these operations, and the many vital structures which it is necessary to divide. But why call ourselves dental surgeons, if unable to perform a greater operation than the extraction of a tooth or a scarifying of the gums? If our knowledge in surgery extends no farther than this, it is very little if any superior to blacksmiths and barbers, who occasionally do the same.

We should also strive to obtain a thorough knowledge of physiological and morbid anatomy, so as to be able to diagnose correctly. We should examine the disease as far as needful for our purpose, and extend our views as far as possible to every thing that has a connection with it. There are many advantages to be derived from it.

1. It will be the means of suggesting to our minds the true nature of the disease.

2. It will enable us to solve any difficulties which may present themselves in its treatment.

3. From our thorough knowledge of the disease, we will be better prepared to treat it according to the principles of our art.

This habit of conceiving clearly and diagnosing correctly, is not to be learned from any set of rules, though these will assist and place us on the right track; but it is observation and practice which must form and establish this habit. We can then, as it were, with ease, grapple with any disease which may present itself, our minds will soon become offended with obscurity and confusion, and restrained from rash judgment. If we adopt this course, we shall treat cases with credit to ourselves, and satisfaction to our patients. Being posted up in every branch directly or indirectly connected with our profession, we shall be prepared to resort to every expedient that science has placed in our hands; and when we fail in any case, shall have the satisfaction of knowing that we have done *all that could be done.*

Every practitioner is aware that in many cases in which he is called upon to prescribe, he has no precedent. In such cases, the skillful practitioner is seldom at a loss; he knows what is dangerous and what is not, what the constitution will bear and what it will not, and governs himself accordingly.

The man who treats symptoms, as such, is an empiric; while the one who labors to remove the cause is a philosopher. The one noticing pain in the head and face, immediately prescribes for neuralgia, while the other patiently challenges every part of the constitution to discover latent inflammation or local irritation, and having found it, proceeds at once to remove it—knowing full well that the effects may be expected to subside, when the cause has been removed. The former is perfectly satisfied that pain and soreness in a sound tooth result from exostosis, and proceeds at once to extract it; while the latter, diligently seeking the cause, ascertains the pain to be sympathetic, and arising from a gravid state of the uterus. This habit is necessary at every step of our professional career. And not even in the simplest cases can we efficiently discharge our duties to our patient and ourselves without it. It is the very basis on which the practice of our art rests. The cultivation of it is raising our profession to the dignity of a noble art; the absence of it would reduce us to the position of charlatans.—*Boston Med. and Surg. Jour.*

